# Body site-specific micro- and lactobiota in genitourinary infections during pregnancy

**DOI:** 10.3389/fcimb.2025.1657715

**Published:** 2025-12-19

**Authors:** Siiri Kõljalg, Epp Sepp, Jelena Štšepetova, Eva-Liina Süüden, Tiia Reimand, Madis Jaagura, Andres Salumets, Reet Mändar

**Affiliations:** 1 Department of Microbiology, Institute of Biomedicine and Translational Medicine, Faculty of Medicine, University of Tartu, Tartu, Estonia; 2 Celvia Competence Centre (CC), Tartu, Estonia; 3 Laboratory of Clinical Microbiology and Mycobacteriology, United Laboratories, Tartu University Hospital, Tartu, Estonia; 4 Institute of Clinical Medicine, Department of Obstetrics and Gynecology, University of Tartu, Tartu, Estonia; 5 Institute of Clinical Medicine, Department of Clinical Genetics, University of Tartu, Tartu, Estonia; 6 Institute of Genomics, University of Tartu, Tartu, Estonia; 7 Division of Obstetrics and Gynecology, Department of Clinical Science, Intervention and Technology, Karolinska Institutet and Karolinska University Hospital, Stockholm, Sweden

**Keywords:** pregnancy, genitourinary microbiota, oral microbiota, rectal microbiota, genitourinary infection

## Abstract

**Background:**

Immunological, metabolic, and hormonal changes occur during pregnancy, which can affect the composition and function of the microbiota in diverse body sites. These changes can influence pregnancy outcomes and the health of the mother and baby. We aimed to characterize the microbiota of pregnant women across various body sites and examine the clinical and population-specific factors associated with its composition. The role of lactobacilli in genitourinary infections during pregnancy was analyzed as well.

**Material and methods:**

One hundred and five pregnant women with gestational age between 15 and 20 weeks were recruited from the Women’s Clinic of Tartu University Hospital, Estonia. Microbiota samples of the cervix (C, *n* = 84), vagina (V, *n* = 85), urine (U, *n* = 105), mouth (M, *n* = 85), and rectum (R, *n* = 84) were analyzed using Illumina NextSeq2000.

**Results:**

Firmicutes was the most common phylum in all investigated locations, with a mean proportion of over 50%. *Lactobacillus* was the most abundant genus in C, V, and U samples (mean proportions of 76%, 77%, and 59%, respectively), while its mean relative abundance was significantly lower in oral (2.8%) and rectal (6.2%) samples. *Lactobacillus iners* was the most frequent *Lactobacillus* species in genitourinary samples, followed by *L.* unidentified (unid)/*crispatus*, *L. jensenii*, and *L. gasseri*. The mean relative abundance of *L. iners* and *L.* unid/*crispatus* in these locations was relatively equal (36% *vs*. 32% in C, 35% *vs*. 33% in V, 25% *vs*. 27% in U). Higher diversity of total urogenital microbiota was associated with chorioamnionitis and metroendometritis at delivery and genitourinary infections during the second half of pregnancy. The latter was associated with lower *Lactobacillus* species diversity in C, V, and U samples. These women also had a lower proportion of *L.* unid/*crispatus* (in C and V samples) and *L. gasseri* (in C, V, and U samples), while they had a higher proportion of *L. iners* in C samples and a higher proportion of other bacteria in C and U samples.

**Conclusions:**

The microbiota of pregnant women is linked to health profile and lifestyle factors and varies in different body regions; however, it is remarkably similar in the cervix and vagina. Higher abundance of *Lactobacillus* species in mid-pregnancy, particularly *L. crispatus* and *L. gasseri*, potentially provides protection against later genitourinary tract infections during pregnancy. This research underscores the importance of microbiota in maternal health and provides a foundation for future studies aimed at developing more effective strategies to support healthy pregnancy.

## 1 Introduction

The human microbiota, the collection of microbes living in various parts of the body, has an important role in maintaining health and influencing disease states (Hou et al., 2022). Differences in microbial composition reflect the unique environment and functions of different body regions (She et al., 2024). Important immunological, metabolic, and hormonal changes occur during pregnancy, which can affect the composition and function of the maternal microbiota (Giannella et al., 2023). Understanding these changes is important because the maternal microbiota can influence pregnancy outcomes and the health of both the mother and the developing fetus (Giannella et al., 2023).

Recent studies have highlighted the importance of the microbiota in various parts of the body of pregnant women, including the vagina, mouth, and rectum (Bisanz et al., 2015; Dunlop et al., 2019; Sparvoli et al., 2020), e.g., changes in the oral microbiota have been linked to preterm delivery (Hong et al., 2023; Park et al., 2024). The microbiota of the genitourinary tract, which includes the cervix, vagina, and urinary tract, is particularly important because it is directly related to reproductive health (Kumar et al., 2023). Microorganisms living in this area form a complex and dynamic ecosystem where different bacteria coexist and interact with each other and with the host and environmental factors (Smith and Ravel, 2017). Healthy microbial communities in the female genitourinary tract are dominated by the genus *Lactobacillus*. Lactobacilli maintain vaginal health by producing lactic acid to keep the pH low, secreting hydrogen peroxide and bacteriocins, adhering to epithelial cells to block opportunistic and pathogenic bacteria, disrupting biofilms, and modulating immune responses to support a balanced microbial environment (Drell et al., 2013; Smith and Ravel, 2017; Punzon-Jimenez and Labarta, 2021). In addition, anti-*Candida* activity of vaginal lactobacilli has recently been associated with their production of acetic acid and exopolysaccharides (Zhang et al., 2023; Pedro and Mira, 2024). *Lactobacillus crispatus*, *L. gasseri*, *L. jensenii*, and *L. iners* are the key species of the vaginal microbiota, each with unique mechanisms and effects on the urogenital tract (Pan et al., 2019). In addition to lactobacilli, the human vagina is colonized by a diverse array of microorganisms that make up the normal microbiota. Disruptions in this microbial ecosystem may result in bacterial vaginosis (BV), aerobic vaginitis (AV), or vulvovaginal candidiasis (VVC), thus significantly impacting the health of the urogenital tract (Chee et al., 2020; Pagar et al., 2024). However, it is not yet fully understood which specific microbial species and how they might impact maternal health during pregnancy and the health of the newborn child.

The objectives of this study were to characterize the microbiota of pregnant women across various body sites and examine the factors influencing microbial diversity and composition. Additionally, it aimed to explore the role of lactobacilli in genitourinary infections during pregnancy. By understanding these interactions, we can gain a deeper insight into the microbiota’s role during pregnancy and develop strategies to enhance maternal and fetal health.

## 2 Materials and methods

### 2.1 Subject enrolment and ethics

Altogether, 105 subjects enrolled at the Tartu University Women’s Clinic between 11 September 2012 and 07 October 2014 were included in the study. The inclusion criteria were as follows: pregnant patient with an indication for amniocentesis (AC) due to a positive second-trimester serum screening test, gestational age between 15 and 20 weeks, and ability to provide written informed consent. Upon enrolment, the subjects were requested to fill in a questionnaire concerning demographic data. Questionnaires concerning pregnancy outcome were completed by using digital medical records and phone or e-mail interviews by an obstetrician after completion of pregnancy.

Subjects consented to participate in a research project, “Gestational microbiota and its association with pregnancy outcome.” Participation in the study was voluntary. The project was reviewed and approved by the Ethics Review Committee on Human Research of Tartu University, Estonia (license no. 215/T-22). Written consent was provided by all subjects.

### 2.2 Sample collection

Study subjects were sampled for microbiota analysis from five different locations: cervix, vagina, urine, mouth, and rectum. Standard procedures were applied to collect the samples for microbiome analyses, and sampling was done under aseptic conditions. The staff were provided with verbal and written instructions as well as sterile sampling devices. Cotton swabs were used by the obstetrician, nurse, or midwife to collect samples from oral, vaginal, cervical, and rectal mucosa that were thereafter placed in the collection tubes. Urine samples were collected by patients who were instructed to wash their hands and genitals with pure water (without soap) and collect the first portion of urine into a plastic container. The specimens were frozen immediately at −20°C and transported to the laboratory within a week, where they were stored at −80°C until molecular analyses. Negative control samples were included in all stages of molecular analyses as a part of standard procedures.

### 2.3 DNA extraction and Illumina sequencing

Bacterial DNA was extracted by using the PureLink™ Microbiome DNA Purification Kit (Invitrogen, USA), using an ELMI Sky Line instrument (ELMI Ltd., Riga, Latvia) according to the manufacturer’s instructions. Extracted DNA samples were quantified with a Qubit fluorometer (Thermo Fisher Scientific, USA) and diluted to 5 ng/mL.

DNA was amplified using primers 16S_F (5′-TCGTCGGCAGCGTCAGATGTGTATAAGAGACAGCCTACGGGNGGCWGCAG-3′) and 16S_R (5′-GTCTCGTGGGCTCGGAGATGTGTATAAGAGACAGGACTACHVGGG TATCTAATCC-3′) for PCR amplification of an approximately 460-bp region within the hypervariable (V3–V4) region of the prokaryotic 16S ribosomal RNA gene (Klindworth et al., 2013). The first PCR mixture contained 12.5 μL of KAPA HiFi HotStart ReadyMix (2X) (Kapa Biosystems, Wilmington, MA), 1 μL of each primer (10 μM), and 5 μL of template DNA (5 ng/μL). The reaction volume was brought to 25 μL with Milli-Q water. PCR conditions were 95°C for 3 min followed by 24 cycles of 95°C for 30 s, 55°C for 30 s, and 72°C for 30 s with a final extension at 72°C for 5 min. The PCR products were purified using a 0.8x solution of AMPure XP Beads (Beckman Coulter, Inc.). The purified products were quantified with Qubit, diluted to 10 ng/μL, and used as a template for indexing PCR. Indexes and sequencing adapters were attached to the PCR products in the indexing PCR, using Illumina Nextera XT dual index primers (Illumina Inc., San Diego, CA). The indexing PCR contained 5 μL of each index primer, 15 μL of KAPA HiFi HotStart ReadyMix (2X), and 5 μL of template DNA (10 ng/μL). The second PCR cycling conditions were 95°C for 3 min followed by 7 cycles of 95°C for 30 s, 55°C for 30 s, and 72°C for 30 s with a final extension at 72°C for 5 min. Indexed PCR products were purified using a 1.9× solution of AMPure XP Beads, quantified, and combined into a final library pool in equimolar concentrations. The library pool was quantified using the Illumina-specific KAPA Library Quant Kit (Kapa Biosystems, Wilmington, MA). Sequencing was carried out on an Illumina NextSeq2000 system using Illumina’s NextSeq reagent kit v3 in paired-end 2 × 300 bp mode.

### 2.4 Statistical analysis

Raw de-multiplexed reads were imported into QIIME2 v2022.2 (Bolyen et al., 2019) and processed using DADA2 (Mane et al., 2009). Denoising was done using default parameters, except for trimming the primer regions and truncating the reverse reads (p-trim-left-f 20, p-trim-left-r 16, trunc-len-f 300, p-trunc-len-r 275). Taxonomy was assigned using a Naive–Bayes classifier trained using Silva 138 reference sequences (Quast et al., 2013). Similar genetic patterns of *L. crispatus* in databases were included to group *L.* unidentified/*crispatus* (*L*. unid/*crispatus*). All non-bacterial reads were excluded from analysis. QIIME2-generated data were further analyzed in R (version 4.1.1) (Team R, 2018).

The alpha-diversity analysis that estimates the species diversity in the single sample was performed applying the richness index (assesses the number of different taxa present in the sample), Shannon index (assesses both richness and evenness of the community), and InvSimpson index (quantifies diversity with an emphasis on dominant taxa, where higher values reflect communities with lower dominance and greater evenness). For different bacteria, their incidence (proportion of samples in a group where a given taxon was detected) and abundance (proportion of the bacterium in a single sample) were analyzed. Associations between lactobacilli in the urogenital tract in mid-pregnancy and the infections in the second half of pregnancy were sought. Comparisons between the study groups were conducted using Fisher’s exact test and chi-squared test. Multiple linear regression was applied including several covariates (age, antibiotic use during pregnancy, living place, education, sexual habits), and false discovery rate correction (FDR) was implemented to reduce the likelihood of false positives due to multiple comparisons.

## 3 Results

### 3.1 Characteristics of the study subjects

Background data of the study subjects are presented in **Table 1**. The median age of the women was 36 (range 17–43) years, and the median sampling time was 18th (range 15–20) week of pregnancy. A quarter (25%) of the subjects received antibiotic treatment during pregnancy before sampling, and one-fifth (21%) experienced infectious disease and 19% genitourinary infections during the second half of pregnancy and/or delivery. Different probiotic products (food and over-the-counter pharmacy products) were used by 72% of pregnant women before sampling. The median duration of pregnancy at delivery was 40 (range 30–42) weeks, and the median offspring birth weight was 3,700 (range 1,978–4,950) g.

**Table 1:** Characteristics of the study subjects.

Characteristics		Mean ± SD	Median (range)	Missing data
Maternal	Age (years)	34.2 ± 5.7	36 (17–43)	0/67
	Higher education	*n* = 17/67 (25%)^a^		0/67
	Previous pregnancies in anamnesis	*n* = 58/67 (87%)^a^		0/67
	Living in town	*n* = 50/67 (75%)^a^		0/67
Pregnancy	Antibiotic treatment during pregnancy before sampling	*n* = 17/67 (25%)^a^		0/67
	Probiotic consumption during pregnancy before sampling	*n* = 48/67 (72%)^a^		0/67
	Taken prescribed medication during pregnancy before sampling	*n* = 12/67 (18%)^a^		0/67
	Duration of pregnancy at sampling (weeks)	17.7 ± 1.1	18 (15–20)	
	Infections during the second half of the pregnancy	*n* = 13/62 (21%)^a^		5/67
	Genitourinary infections during the second half of pregnancy and delivery	*n* = 10/62 (19%)^a^		5/67
	Threatened miscarriage	*n* = 14/67 (21%)^a^		
Birth	Duration of pregnancy at delivery (weeks)	39.7 ± 2.2	40 (30–42)	
	Chorioamnionitis/metroendometritis	*n* = 4/62 (7%)^a^		5/67
	Timely delivery	*n* = 58/62 (94%)^a^		5/67
	Cesarean section	*n* = 24/61 (39%)^a^		6/67
Child	Birth weight (g)	3,635.3 ± 511.4	3,700 (1,978–4,950)	
	Birth weight >4,000 g	*n* = 11/61 (18%)^a^		6/67

A total of 105 pregnant women were studied, with 67 of these women having medical and questionnaire data available for further analysis.

a Data are given as the number (percentage) of patients.

### 3.2 Microbial communities in different body locations

**Figure 1:**
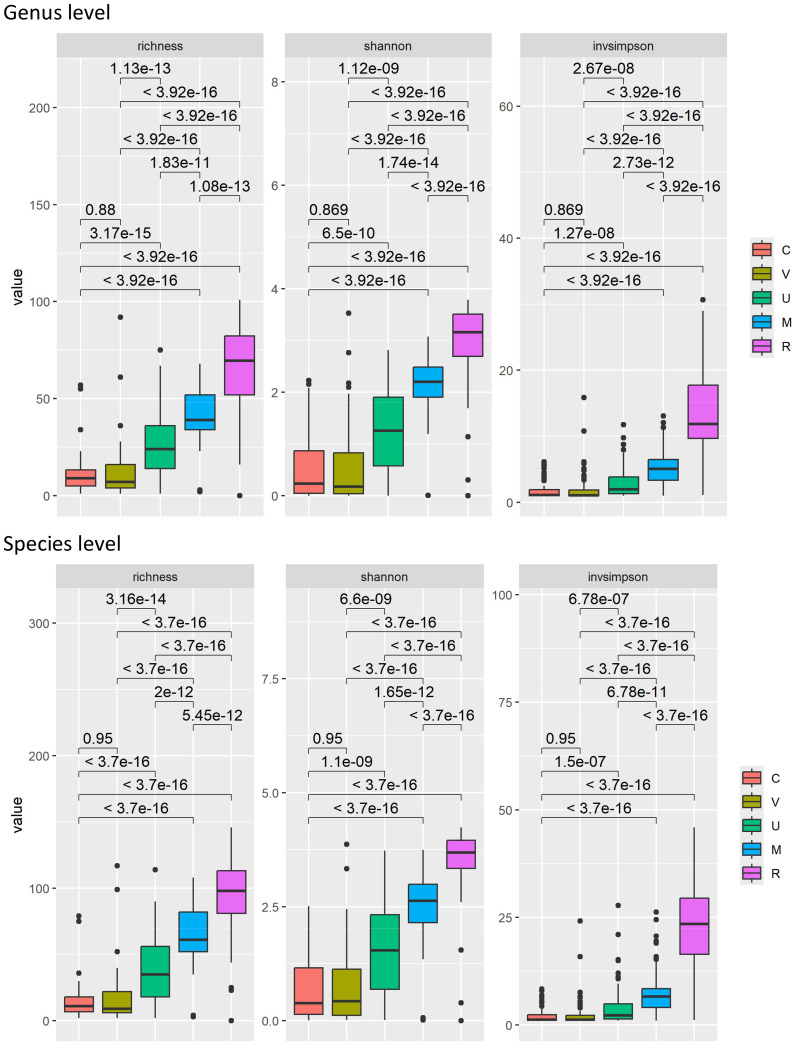
Richness and diversity indices of the analyzed samples at the genus and species levels. FDR-adjusted *p*-values are indicated. Sample sources: cervix (C), vagina (V), urine (U), mouth (M), and rectum (R).

In total, 443 samples from 105 women were available for microbiota analysis, including 85 vaginal (V), 84 cervical (C), 105 urine (U), 85 oral (M), and 84 rectal (R) samples. As expected, the rectal communities were more diverse than urogenital tract communities, with oral communities falling in between (**Figure 1**).

**Figure 2:**
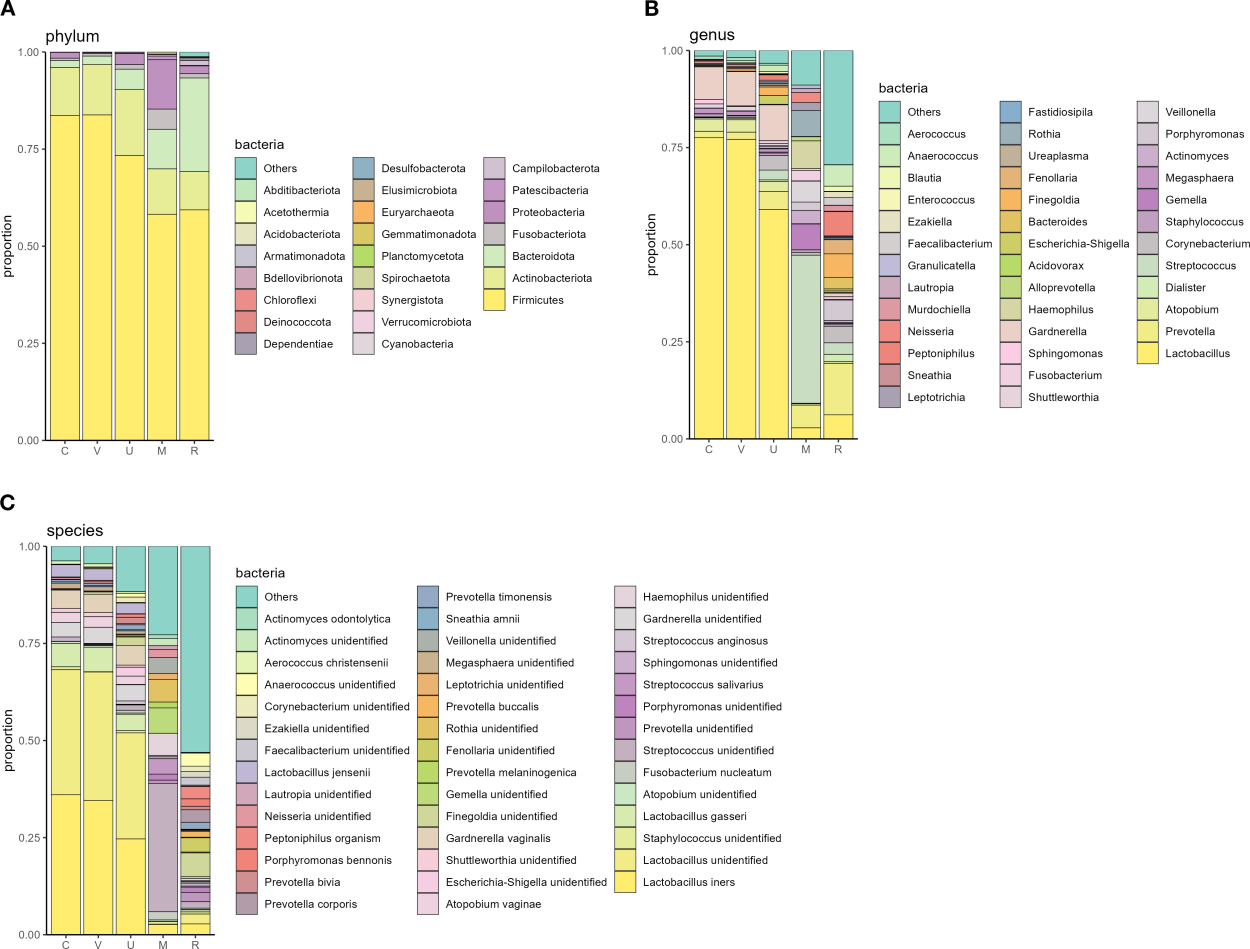
Mean proportions of top phyla **(A)**, genera **(B)**, and species **(C)** found in the analyzed samples. Sample sources: cervix (C), vagina (V), urine (U), mouth (M), and rectum (R).

Firmicutes (Bacillota) was the most common microbial phylum found in all investigated locations, with a mean proportion over 50% (**Figure 2A**), followed by Actinobacteriota and Bacteroidota in cervical, vaginal, and urine samples. In oral samples, Firmicutes were followed by Proteobacteria, Actinobacteriota, and Bacteroidota, and in rectal samples, Firmicutes were followed by Bacteroidota and Actinobacteriota (**Figure 2A**).

The most predominant bacterial genus found in C, V, and U was *Lactobacillus*; in the mouth, *Streptococcus*; and in the rectum, *Prevotella*. The mean relative abundance of *Lactobacillu*s was approximately three-quarters in cervical and vaginal samples and exceeded 50% in urine samples (76.4%, 76.9%, and 58.9%, respectively), followed by *Gardnerella* (8.2%, 8.8%, and 9.2%, respectively). In the cervix, the genera found were *Atopobium*, *Prevotella*, and *Megasphaera* (3.1%, 1.7%, and 1.4%, respectively); in the vagina, *Atopobium, Prevotella*, and *Shuttleworthia* (3.2%, 1.9%, and 1.1%, respectively); and in urine, *Prevotella*, *Corynebacterium*, and *Atopobium* (4.6%, 3.7%, and 2.5%, respectively; **Figure 2B**).

**Figure 3:**
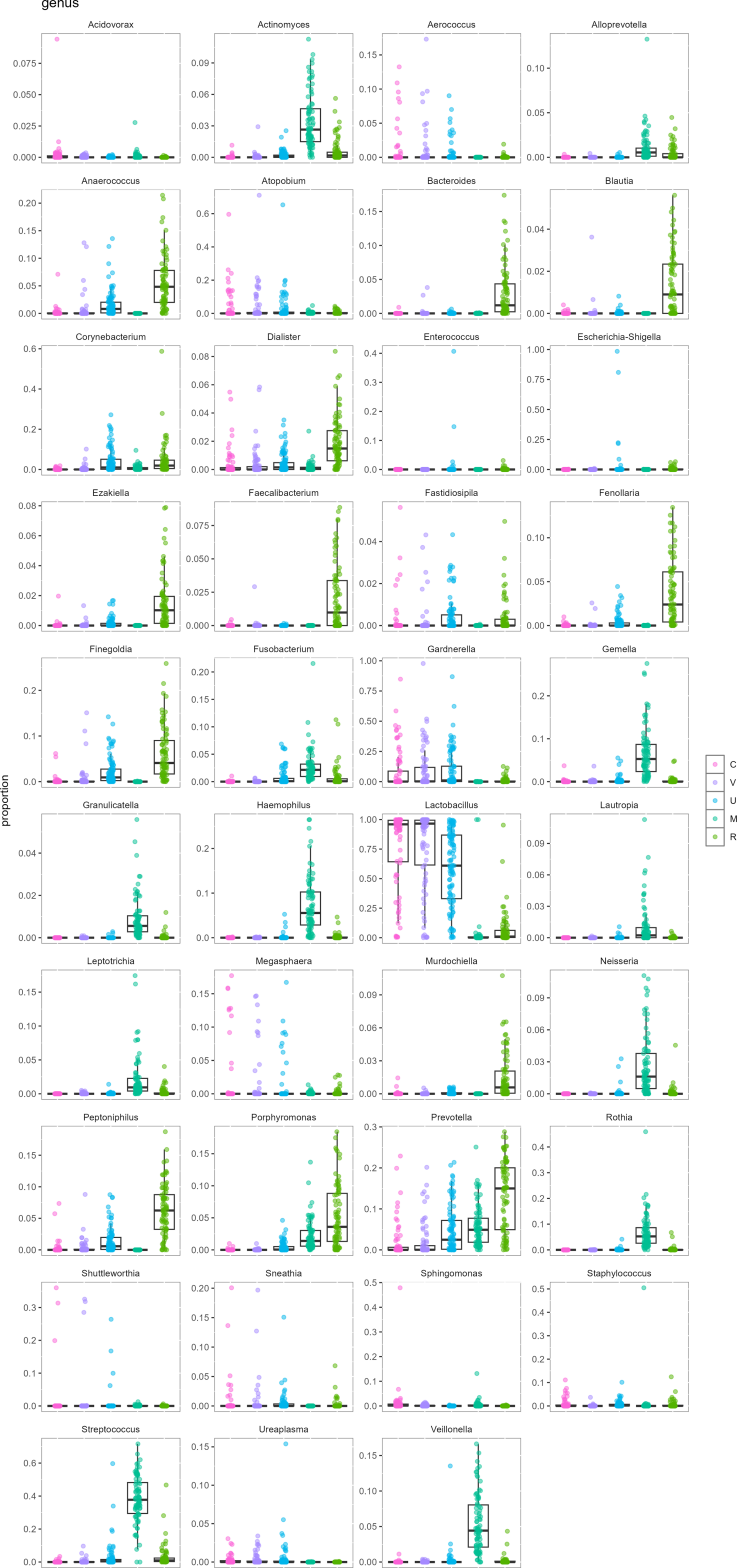
Distribution of top genera in different sample sources. Order of sample sources: cervix (C), vagina (V), urine (U), mouth (M), and rectum (R).

The genus *Streptococcus* predominated in oral samples (mean proportion per sample constituted 37.8%), followed by *Haemophilus* (7.1%), *Rothia* (6.6%), *Gemella* (6.6%), and *Prevotella* (5.8%). In rectal samples, the highest average proportion was found for *Prevotella* (13.2%), followed by *Peptoniphilus* (6.2%), *Lactobacillus* (6.2%), *Finegoldia* (6.1%), and *Anaerococcus* (5.5%; **Figure 2B**). **Figure 3** illustrates the distribution of top genera between different sample sources.

### 3.3 Microbiome richness and diversity depending on subject-related factors

We found higher microbiome diversity (**Table 2**) in cervical samples at the first half of the pregnancy in women living in the countryside compared to those living in towns (species level Shannon index *p* = 0.02 and InvSimpson index *p* = 0.02), and we also found richness in women who had chorioamnionitis or metroendometritis at the time of delivery compared to women with no aforementioned infections (genus-level richness index *p* = 0.04 and species-level richness index *p* = 0.05). Higher richness and diversity in both genus and species levels in vaginal samples was found in women having genitourinary (GU) infections during the second half of pregnancy compared to no GU infection episodes (genus-level richness index *p* = 0.03, Shannon index *p* = 0.02, InvSimpson index *p* = 0.02, species-level richness index *p* = 0.04, Shannon index *p* = 0.03, InvSimpson index *p* = 0.04).

**Table 2:** Diversity of microbial communities in different locations (urine, oral, vaginal, cervical, rectal) depending on influencing factors.

Sample source	Subject characteristics	Diversity level	Higher Richness	Higher Shannon index	Higher InvSimpson index
			*p*-value	*p*-value	*p*-value
Cervix	Living in the countryside	Genus	ns	ns	ns
		Species	ns	0.023	0.016
	Chorioamnionitis/metroendometritis	Genus	0.040	ns	ns*
		Species	0.047	ns*	ns**
Vagina	Genitourinary infection during the second half of pregnancy	Genus	0.025	0.015*	0.02
		Species	0.038	0.033*	0.043
Urine	Education secondary and primary	Genus	0.048	ns	ns
		Species	0.025	ns	ns
	Inflammation during the second half of pregnancy	Genus	ns	ns	ns
		Species	0.019	ns	ns
Mouth	No previous abortion	Genus	ns	ns	ns
		Species	0.043	ns	ns
	No medications used during pregnancy	Genus	0.018	ns	ns
		Species	0.010	ns	ns
	No antibiotics during pregnancy	Genus	0.013	ns	ns
		Species	0.009	ns	ns
	Premature birth	Genus	ns**	0.015	0.015**
		Species	ns**	ns	ns***

Asterisks indicate the associations that remained significant or borderline after multiple linear regression analysis, and the FDR-adjusted *p*-values are presented as follows: *p < 0.1, **p < 0.05, ***p < 0.01.

ns, not significant.

Higher richness in urine samples was found in women with secondary and basic education compared to women with higher education (genus-level richness index *p* = 0.05 and species-level Shannon index 0.03) and in women with inflammation during the second half of pregnancy compared to women with no inflammation (species-level richness index *p* = 0.02).

Oral microbiome richness was higher in cases of no previous abortion, no medications used during pregnancy, and no antibiotic consumption during pregnancy compared to the presence of all these characteristics (species richness index *p* = 0.04; genus richness index *p* = 0.02 and species richness index *p* = 0.01; genus richness index *p* = 0.01 and species richness index *p* = 0.01, respectively). In cases of premature birth, microbial diversity in oral samples was also higher compared to timely delivery (genus Shannon index *p* = 0.02, genus InvSimpson index *p* = 0.02).

No statistically significant differences in diversity were found depending on women’s age, previous pregnancies, risk of miscarriage during pregnancy, group-B *Streptococcus* prophylaxis, delivery method (normal *vs*. assisted), or lower birth weight (under 3,000 *vs*. over 3,000 g).

We thereafter applied multiple linear regression that included several covariates (age, antibiotic use during pregnancy, living place, education, sexual habits). False discovery rate correction (FDR) was implemented to reduce the likelihood of false positives due to multiple comparisons (**Supplementary Figure S1**). The above-described associations that remained significant or borderline after multiple linear regression analysis were indicated with asterisks in **Table 2**. In addition, this analysis revealed a significant association between rectal microbiome and use of prescribed medications during pregnancy (species-level Shannon index *p* = 0.047, genus-level Shannon index *p* = 0.059) and some borderline associations, including the link between vaginal microbiome and chorioamnionitis/metroendometritis (species-level Shannon index *p* = 0.070, species-level InvSimpson index *p* = 0.074, genus-level InvSimpson index *p* = 0.078), between cervical microbiome and threatened miscarriage (genus-level InvSimpson index *p* = 0.062), between vaginal microbiome and birth weight (genus-level InvSimpson index *p* = 0.061), and between urine microbiome and duration of pregnancy at delivery (genus-level InvSimpson index *p* = 0.084) (**Supplementary Figure S1**).

### 3.4 Lactobacilli in different sample sources depending on subject-related factors

**Figure 4:**
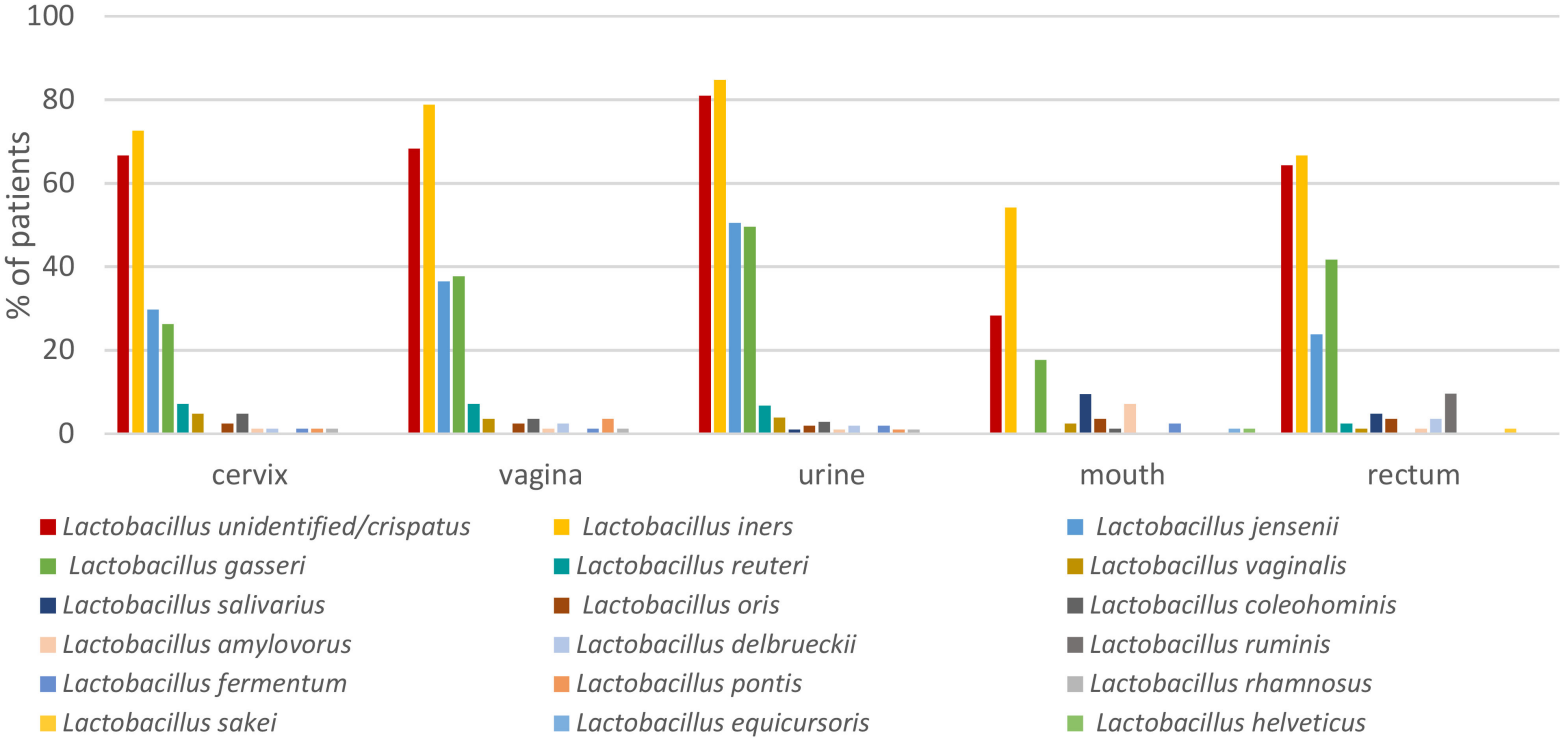
Incidence of different *Lactobacillus* species from different sample sources.

*Lactobacillus iners* had the highest incidence among *Lactobacillus* spp. in genitourinary samples (72.6% in C, 78.8% in V, 84.8% in U samples) (**Figure 4**), followed by *L.* unid/*crispatus* found in 66.7%, 68.2%, and 81.0% of the studied samples, respectively. *Lactobacillus jensenii* was in third place in the cervix and urine, while *L. gasseri* was found in the vagina (29.8%, 50.5% and 37.6%, respectively). The mean relative abundance of *L. iners* and *L.* unid/*crispatus* in these locations was quite equal (35.7% *vs*. 31.8% in C, 34.5% *vs*. 33.0% in V, 24.6% *vs*. 27.2% in U, respectively) (**Figure 2C**).

In oral samples, the most frequent *Lactobacillus* was *L. iners*, followed by *L.* unid/*crispatus*, *L. gasseri*, *L. salivarius*, and *L. amylovorus* (found in 54.1%, 28.2%, 17.6%, 9.4%, and 7.1% of pregnant women, respectively). In rectal samples, the most frequent *Lactobacillus* was also *L. iners*, followed by *L.* unid/*crispatus*, *L. gasseri*, *L. jensenii*, and *L. ruminis* (found in 66.7%, 64.3%, 41.7%, 23.8%, and 9.5% subjects, respectively) (**Figure 4**). The mean relative abundance of lactobacilli was significantly lower in the mouth (2.8%) and rectum (6.2%) in comparison with the urogenital tract (**Figure 2B**).

*Lactobacillus iners* had higher relative abundance in vaginal samples among higher educated pregnant women compared to women with secondary or basic education, as well as in cervical samples in case of timely delivery compared to premature delivery (*p* = 0.04 and *p* = 0.05, respectively).

At the same time, *L. gasseri* had higher relative abundance in cervical and vaginal samples of subjects over 36 years compared to younger women (*p* = 0.01 and *p* < 0.01, respectively) and in cervical samples of women with babies >4,000 g compared to those of women with babies <3,000 g at delivery (*p* = 0.01). *Lactobacillus jensenii* had higher relative abundance in cervical samples of subjects >36 years of age compared to younger ones (*p* = 0.02). In rectal samples, *L. jensenii* was found in a higher proportion in women >36 years compared to younger subjects and without chorioamnionitis or metroendometritis (*p* = 0.03 and *p* = 0.01, respectively). *Lactobacillus oris* was found in a higher incidence in women experiencing chorioamnionitis or metroendometritis compared to women with no aforementioned infections (*p* = 0.015) in rectal samples.

### 3.5 Association of lactobacilli in mid-pregnancy with infections in the second half of pregnancy

To estimate the possible influence of microbiota on subsequent infections, 62 pregnant women for whom we had relevant information were divided into two subgroups according to the presence (group I; *n* = 10) or absence (group II; *n* = 52) of genitourinary infections during the second half of pregnancy or delivery. Infections encountered were metroendometritis (*n* = 3), chorioamnionitis plus urinary tract infection (*n* = 1), UTI (*n* = 5), and *Candida* colpitis (*n* = 1).

**Figure 5:**
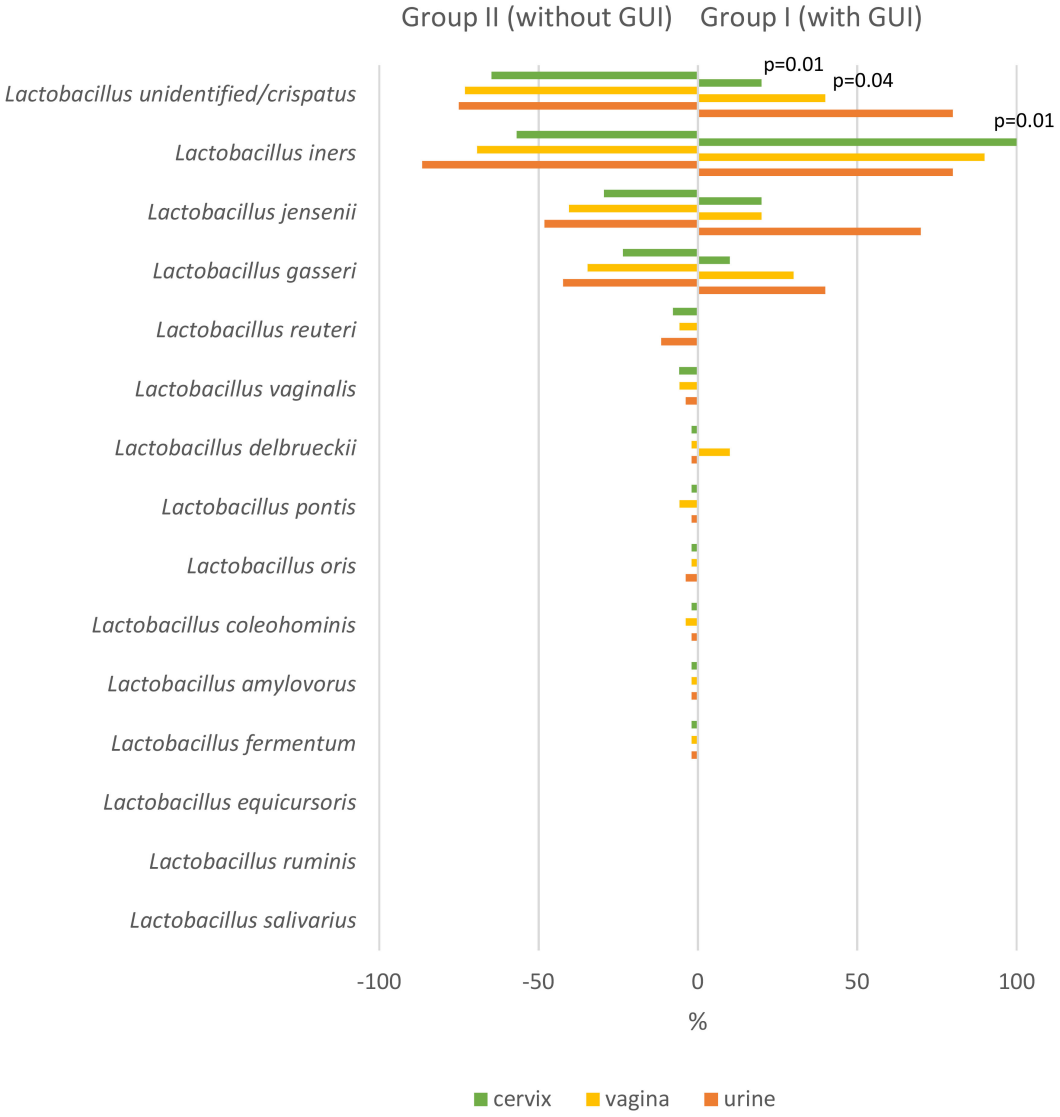
Incidence of different *Lactobacillus* species in the cervix, vagina, and urine among mid-pregnancy women with (group I) and without (group II) subsequent genitourinary infections (GUIs) during the second half of pregnancy.

Group I women (having infections) had lower *Lactobacillus* spp. diversity in C, V, and U samples but not in M and R samples compared to group II women. The number of different *Lactobacillus* species was 4 *vs*. 12 (*p* = 0.01) in C, 5 *vs*. 12 (*p* = 0.03) in V, and 4 *vs*. 12 (*p* = 0.01) in U samples, while these were 4 *vs*. 8 (*p* = 0.28) in M and 8 *vs*. 7 (*p* = 1.0) in R samples, respectively. The respective median different *Lactobacillus* species numbers concerning individual subjects in group I *vs*. group II were 1.5 *vs*. 2.0 in C, 1.9 *vs*. 2.4 in V, 2.7 *vs*. 2.8 in U, 1.0 *vs*. 1.2 in M, and 2.7 *vs*. 2.6 in R samples, and there were no statistically significant differences between the groups. In addition, seven *Lactobacillus* species, namely, *L. amylovorus*, *L. coleohominis*, *L. fermentum*, *L. oris*, *L. pontis*, *L. reuteri*, and *L. vaginalis*, were found in the urogenital tract only in women in group II but not in group I (**Figure 5**).

*Lactobacillus* unid/*crispatus* was the most frequently found *Lactobacillus* in group II detected from 65% of C and 73% of V samples. *Lactobacillus iners*, on the other hand, was the most frequently found *Lactobacillus* in group I, detected from 100% of C and 90% of V samples. In group I U samples, *L.* unid*/crispatus* and *L. iners* were found equally among 80% of the studied individuals. *Lactobacillus iners* was more frequently found in C samples in group I subjects compared to group II subjects (100% *vs*. 57%, respectively; *p* = 0.01). *Lactobacillus* unid/*crispatus* was more frequently found in group II subjects compared to group I subjects from C (64% *vs*. 20%, *p* = 0.01) and V samples (73% *vs*. 40%, *p* = 0.04) (**Figure 5**).

**Figure 6:**
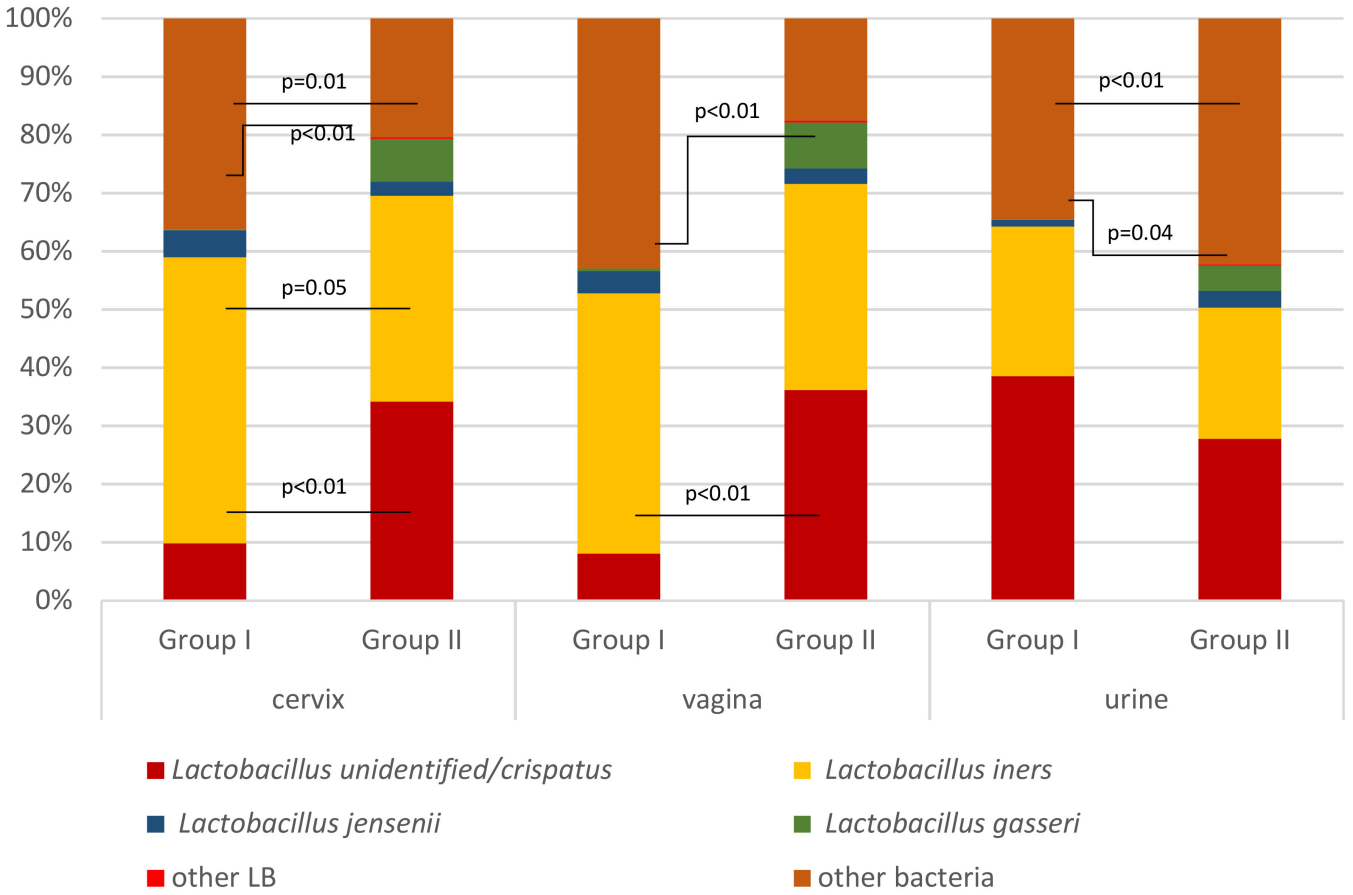
Mean proportion of different *Lactobacillus* species and other bacteria in the cervix, vagina, and urine among mid-pregnancy women with (group I) and without (group II) subsequent genitourinary infections during the second half of pregnancy.

The mean proportion (abundance) of each species in the community was analyzed as well (**Figure 6**). The mean proportion of *L. iners* was higher in group I compared to group II in C (49.1% *vs*. 35.3%, *p* = 0.05, respectively), but not in V, U, M, and R samples. The mean proportion of *L.* unid/*crispatus* was higher in group II in C and V samples (34.2% *vs*. 9.8%, *p* < 0.01, and 36.1% *vs*. 8.0%, *p* < 0.01, respectively), but not in U, M, and R samples. Also, that of *L. gasseri* was higher in group II compared to group I in C (7.3% *vs*. 0.1%, *p* < 0.01, respectively) and V (7.9% *vs*. 0.5%, *p* < 0.01, respectively) but in group I compared to group II in U samples (4.4% *vs*. 0.04%, *p* = 0.04, respectively). The mean proportion of other bacteria compared to lactobacilli was higher in group I compared to group II in C and U samples (36.3% *vs*. 20.4%, *p* = 0.01, and 42.9% *vs*. 17.6%, *p* < 0.01, respectively).

## 4 Discussion

Our study provides a comprehensive analysis of the microbiota of pregnant women in different body regions, highlighting significant differences in microbial composition and diversity associated with demographic and health factors. Our results agree with previous studies, emphasizing the critical role of microbiota in maternal health and pregnancy outcomes. Diversity and richness of the microbial communities tended to be associated with several host- and environment-related factors. Specific *Lactobacillus* species dominated in different body regions, and their abundance was associated with maternal age, education level, timeliness of delivery, and infectious conditions. We found that pregnant women who developed genitourinary tract infections in the second half of pregnancy had significantly lower diversity and abundance of beneficial *Lactobacillus* species in mid-pregnancy, particularly *L. crispatus* and *L. gasseri*, while *L. iners* was more dominant among those infected, suggesting a potential protective role for certain lactobacilli species against later infections.

### 4.1 Microbiota of pregnant women

The microbiota of pregnant women in different body locations, mainly the vagina and gut, but also the mouth, has been described in several studies (Bisanz et al., 2015; Dunlop et al., 2019; Di Simone et al., 2020). Other body locations have been less researched, while comprehensive studies that simultaneously analyze multiple key body areas with microbial habitats remain largely absent. Our study stands out among the microbiota research conducted on pregnant women as we addressed the microbiota in several body areas and samples such as the cervix, vagina, urine, mouth, and rectum. We revealed that cervical and vaginal microbiota are remarkably similar, both being relatively similar to urinary microbiota samples. In all these locations, lactobacilli predominate. Oral and rectal microbiota differ significantly from each other and from genitourinary microbiota. The presence of *Streptococcus* in the mouth and *Prevotella* in the rectum further reflects different microbial communities that have adapted to different body environments (Burcham et al., 2020; Prasoodanan et al., 2021). Yet, Firmicutes was the dominant microbial phylum in all body regions examined, which is consistent with the available literature (Perez-Carrasco et al., 2021; Hou et al., 2022) including our previous studies (Mändar et al., 2015; Drell et al., 2017; Mändar et al., 2023). Firmicutes were followed by Actinobacteriota in cervical, vaginal, and urine samples; by Proteobacteria, Actinobacteriota, and Bacteroidota in oral samples; and by Bacteroidota and Actinobacteriota in rectal samples.

Higher microbial diversity and richness in the intestinal tract have long been recognized as favorable for health characteristics (Hills et al., 2019; Manor et al., 2020; Sepp et al., 2022). The gut microbiota is intricately connected to the female reproductive tract microbiota, influencing its composition and function through various mechanisms, including immune modulation and metabolic interactions, which can impact susceptibility to infections (Amabebe and Anumba, 2020). At the same time, high microbial diversity in the genitourinary tract is associated with poor reproductive outcome, like risk for preterm birth (Di Simone et al., 2020; Baud et al., 2023), as well as a high frequency of genital tract and urinary tract infections (France et al., 2022). In our study, higher microbial richness in the GU tract in the first half of the pregnancy was found in women with GU infections, especially chorioamnionitis or metroendometritis. Alterations in the oral microbiota have been associated with preterm birth (Hong et al., 2023; Park et al., 2024). We found that in cases of premature birth, microbial diversity in the mothers’ mouth was higher compared to women who gave birth in a timely delivery. We also found higher microbial richness in the mouths of women not receiving prescribed medications and antibiotics, which indicates the selective influence of medications on oral microbes. However, all these associations should be interpreted with caution due to the cross-sectional nature of the study.

### 4.2 Lactobiota of pregnant women

As expected, *Lactobacillus* was found to be the most predominant bacterial genus in genitourinary samples (Mändar et al., 2015; Mändar et al., 2023). Their dominance emphasizes their importance in maintaining vaginal health and preventing infections. *Lactobacillus crispatus* was found more often in cases of pregnancies proceeding without GU infections. The protective role of *L. crispatus* has also been emphasized previously (Petrova et al., 2017; Mändar et al., 2023). In a recent study (Tan et al., 2025), the abundance and species composition of vaginal lactobacilli were compared between women with recurrent miscarriage and controls. This study employed a distinct analytical approach, 2bRAD-M, which enables high-resolution species-level identification and functional profiling. Despite differences in methodology and subject cohort, both studies highlighted the beneficial role of lactobacilli, particularly *L. crispatus*. A recent review (Gao et al., 2024) examined the role of vaginal and endometrial microbiomes in women with repeated implantation failure and recurrent pregnancy loss. Consistently, a higher prevalence of *L. crispatus* was associated with favorable reproductive outcomes, whereas *L. iners* and other dysbiotic profiles were linked to adverse events.

In addition, in our study, women with GU infections during the second half of the pregnancy had fewer different *Lactobacillus* species in the GU tract at the first half of pregnancy in the present study. Pregnant women without subsequent GU infections also had seven *Lactobacillus* species not found in the GU infection group in C, V, and U samples. Though the high overall microbial diversity in the vagina is associated with unfavorable outcomes of pregnancy (Baud et al., 2023), high *Lactobacillus* species diversity seems to offer protection against GU infections. *Lactobacillus gasseri* and *L.* unid*/crispatus* proportions were higher, and the proportion of all the other bacteria compared to lactobacilli was lower in the genital tract in women without subsequent GUIs in our study; on the other hand, *L. iners* was found more frequently in women with the following respective infections. Again, all the associations with different lactobacilli revealed in this project should be interpreted with caution due to the cross-sectional nature of the study.

Previous studies have revealed that *L. gasseri* and *L. crispatus* play key roles in maintaining a healthy vaginal microbiota (Petrova et al., 2015). These species are known for their ability to produce high amounts of lactic acid, which helps maintain a low vaginal pH, creating an environment hostile to pathogenic bacteria (Ravel et al., 2011; Mändar et al., 2023). *Lactobacillus gasseri* also produces bacteriocins, which are antimicrobial peptides that inhibit the growth of harmful microorganisms (Zhang et al., 2024). In addition, *L. gasseri* has been shown to adhere well to vaginal epithelial cells, which helps it to compete with and prevent the colonization of mucosa by pathogens (Zhang et al., 2024). Its presence is associated with a lower risk of bacterial vaginosis and other vaginal infections (Qi et al., 2023). This makes *L. gasseri* an important component of probiotic therapy aimed at restoring and maintaining vaginal health. *Lactobacillus iners* is also an important component of the vaginal microbiota, playing a crucial role in maintaining vaginal health (Zheng et al., 2021). However, unlike other *Lactobacillus* species, *L. iners* has a smaller genome, which affects its metabolic capabilities and adaptability (Petrova et al., 2017; Vaneechoutte, 2017). *Lactobacillus iners* has been associated with less protection against vaginal dysbiosis than other *Lactobacillus* species (Petrova et al., 2015). Its presence is often associated with conditions such as bacterial vaginosis, sexually transmitted infections, and adverse pregnancy outcomes (Vaneechoutte, 2017; Zheng et al., 2021). Despite these seemingly negative associations, *L. iners* contributes to overall microbial balance by producing lactic acid, which helps maintain an acidic vaginal pH (Zheng et al., 2021).

Identification of specific *Lactobacillus* species associated with favorable pregnancy outcomes will provide valuable information for the development of targeted probiotic therapies (Qi et al., 2023; Zhang et al., 2024). In addition, monitoring the diversity and composition of lactobiota and full microbiota in different body locations of pregnant women may be a predictive tool to identify pregnant women at risk of adverse outcomes, allowing for timely intervention.

### 4.3 Limitations of the study

There are some limitations of our study. Although the sample size is significant, it may not fully capture the diversity of the pregnant population. Future studies involving larger and more diverse cohorts are needed to confirm our results. The study could also have benefited from the inclusion of a control group of non-pregnant women. In addition, vaginal microbiota is dynamic and undergoes changes throughout the gestational period. During the second half of pregnancy, the vaginal microbiota is typically marked by low microbial diversity and a strong dominance of *Lactobacillus* species that helps maintain vaginal health and reduces the risk of ascending infections that could compromise pregnancy outcomes (Mändar et al., 1995; Baud et al., 2023). Therefore, longitudinal studies monitoring microbiota changes during the entire pregnancy and their direct effects on maternal and fetal health are needed to establish causal relationships with pregnancy outcome.

## 5 Conclusion

Taken together, our study shows that the microbiota of pregnant women is linked to health profile and lifestyle factors and varies in different body regions; however, it is remarkably similar in the cervix and vagina. The higher abundance of lactobacilli in mid-pregnancy—particularly *L. crispatus* and *L. gasseri*—potentially provides protection against later genitourinary tract infections. By examining a diverse range of biosamples from different body sites, we have improved our understanding of the intricate relationship between microbiota and pregnancy outcome. This research underscores the importance of microbiota in maternal health and provides a foundation for future studies aimed at developing more effective strategies to support healthy pregnancy.

## Data Availability

The original contributions presented in the study are publicly available. This data can be found here: NCBI/PRJNA1380350.
