# Supplementary material for: Body site-specific micro- and lactobiota in genitourinary infections during pregnancy
Source: Front Cell Infect Microbiol. 2025 Dec 19;15:1657715. doi: 10.3389/fcimb.2025.1657715 (PMC12757402; doi:10.3389/fcimb.2025.1657715)

Supplementary Figure S1. Richness and diversity indices of the analysed samples after multiple linear regression that included several covariates (age, antibiotic use during pregnancy, living place, education, sexual habits). FDR-adjusted *p* values are indicated. Data are visualised on genus and species level. Sample sources: cervix (C), vagina (V), urine (U), mouth (M), rectum (R)

Living in town

Genus

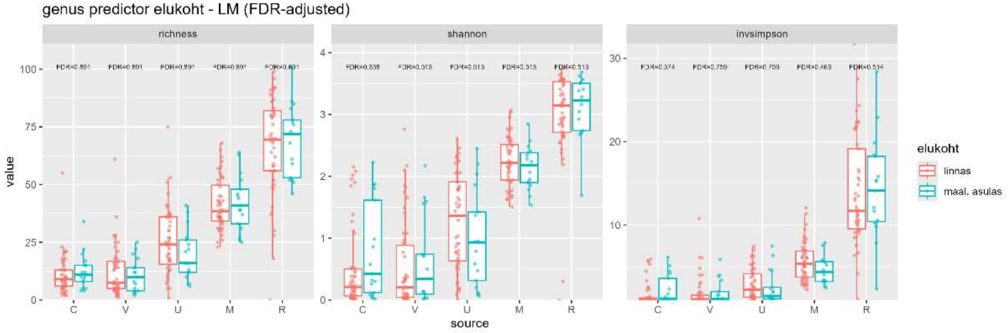

Species

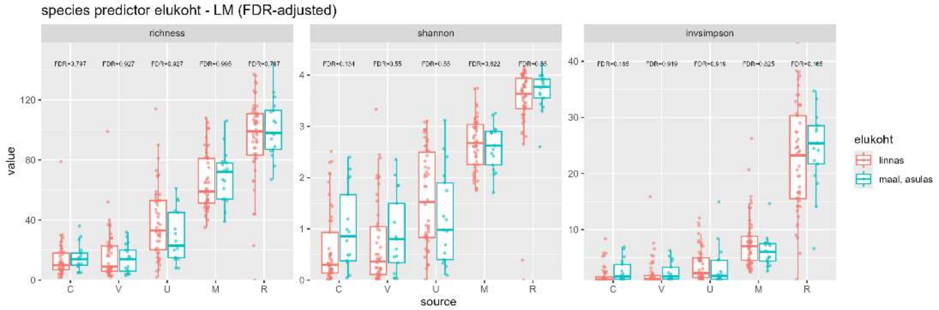

Education

Genus

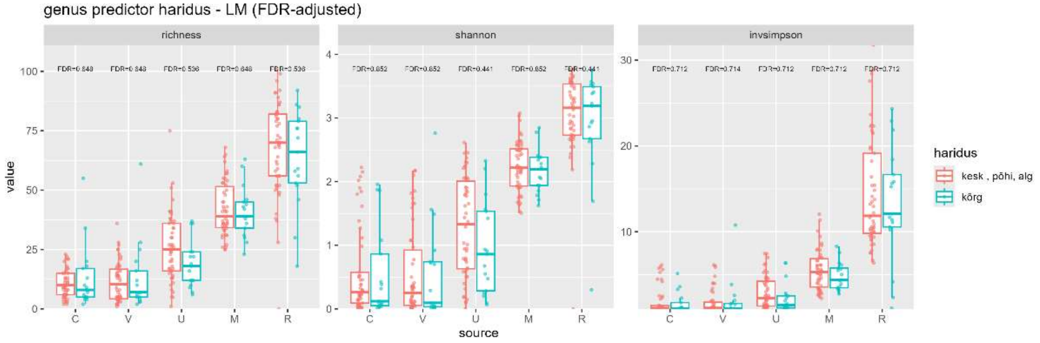

Species

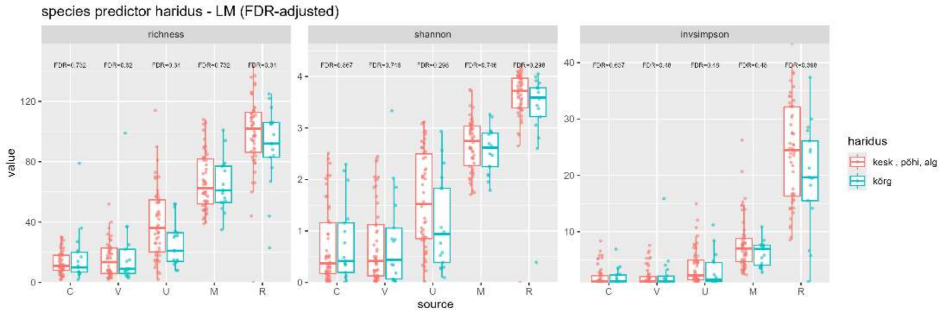

Previous pregnancies in anamnesis

Genus

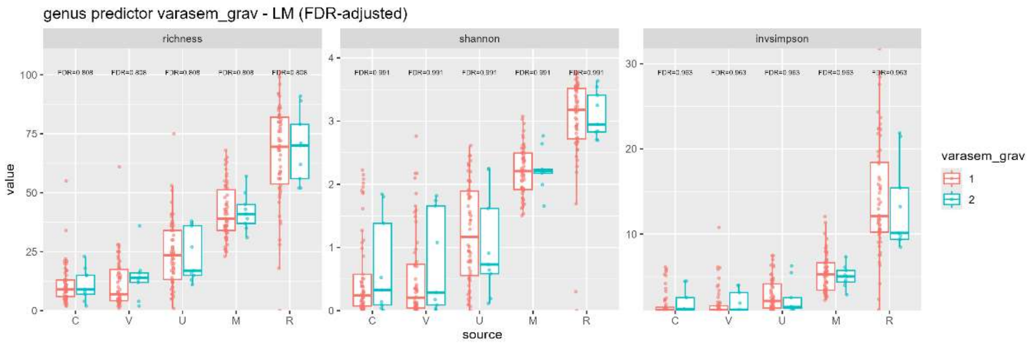

Species

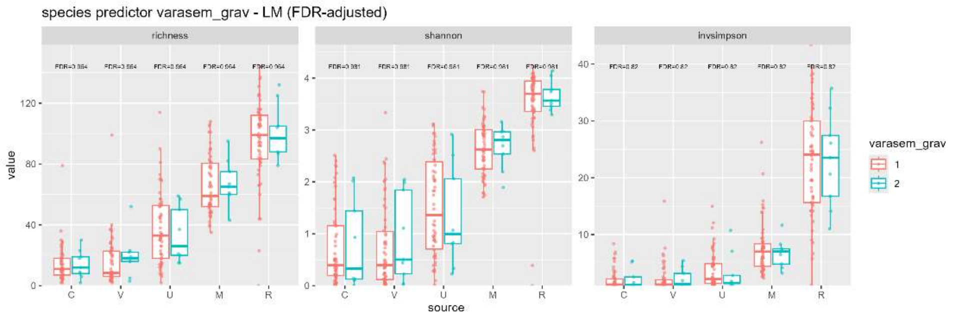

Antibiotics during pregnancy

Genus

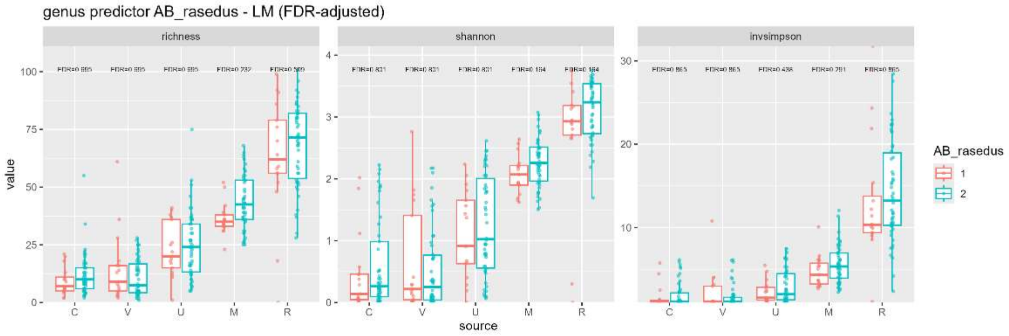

Species

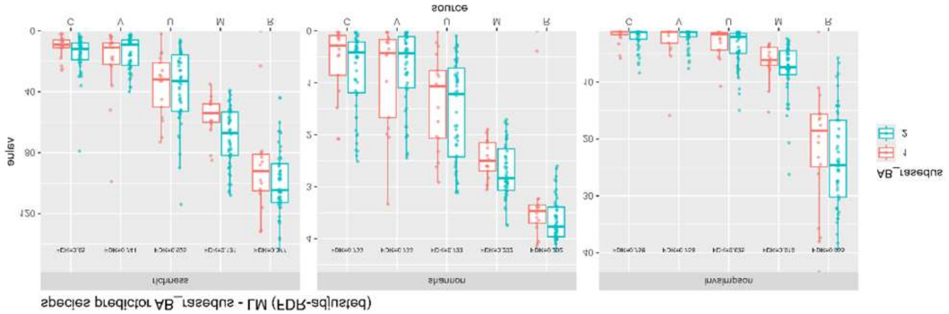

Other prescribed drugs during pregnancy

Genus

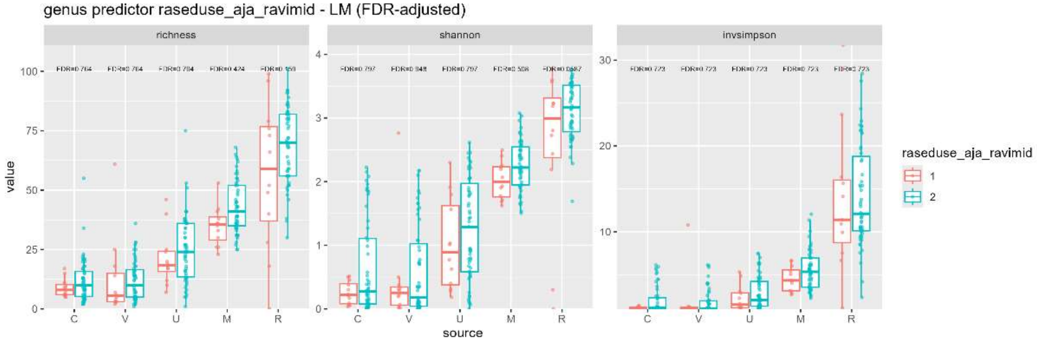

Species

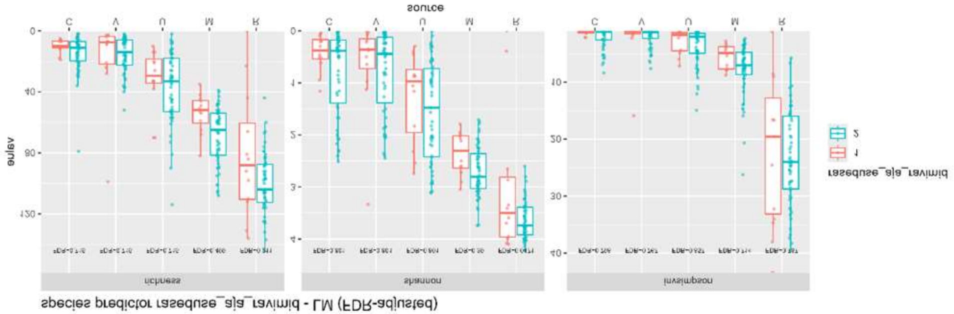

Threatened miscarriage

Genus

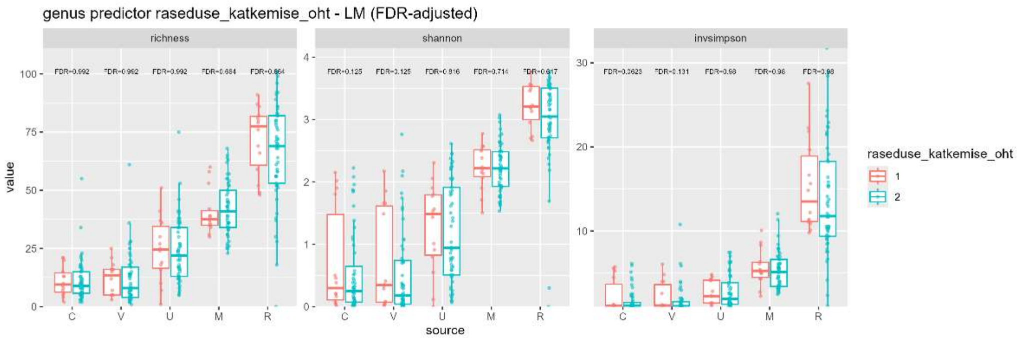

Species

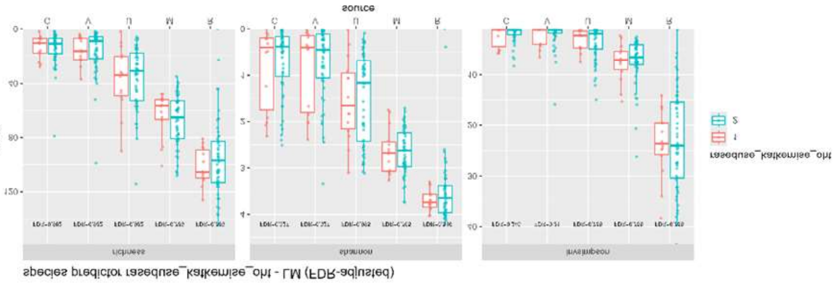

Genitourinary infections during 2nd half of pregnancy

Genus

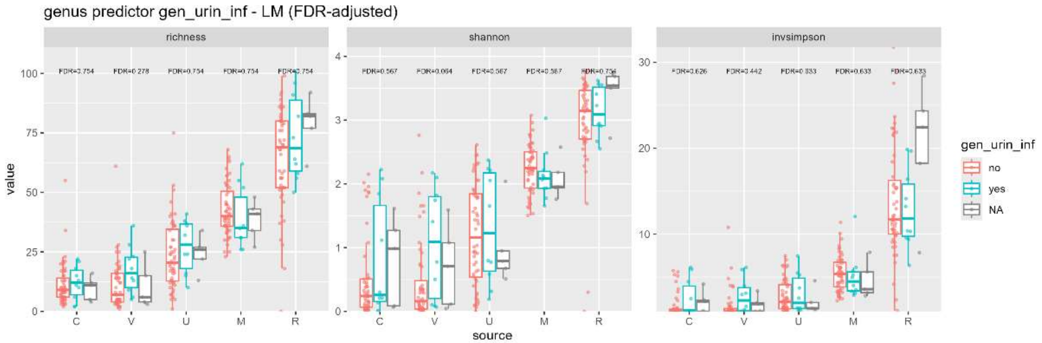

Species

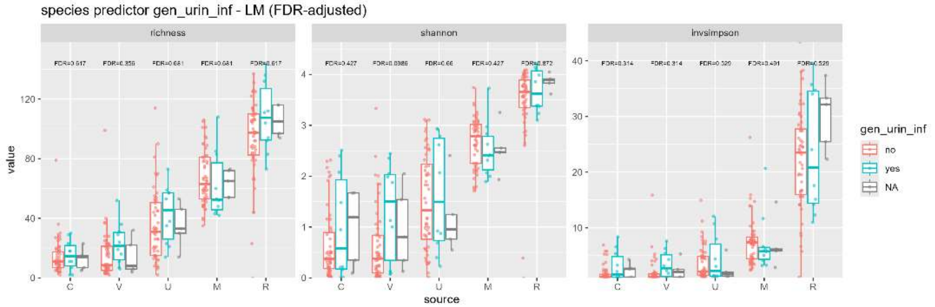

Chorioamnionitis or metroendometritis

Genus

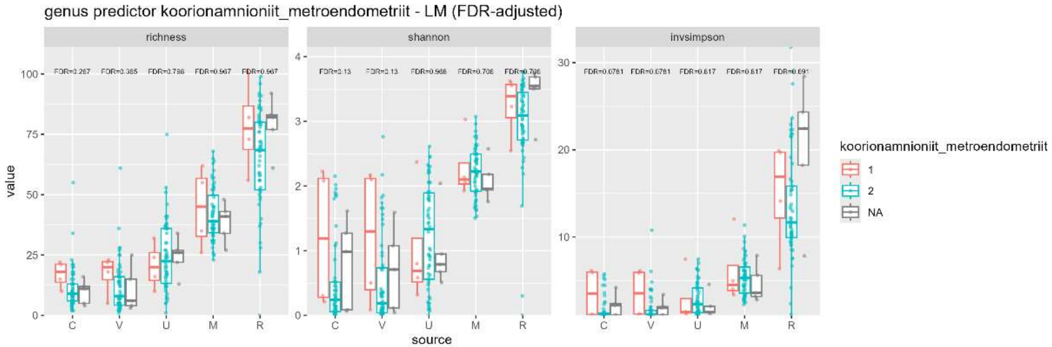

Species

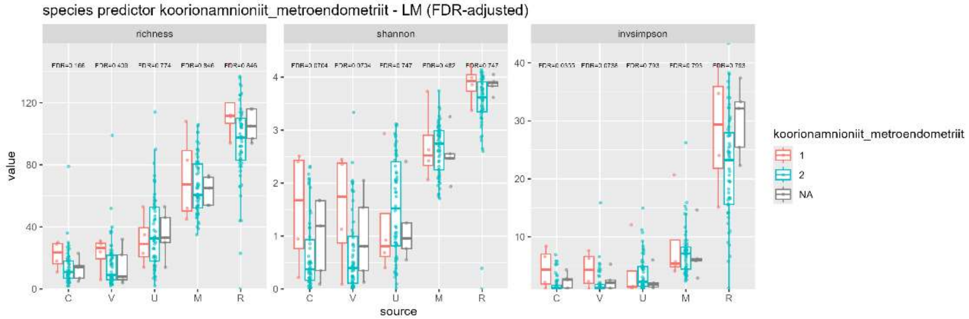

Birth weight (g)

Genus

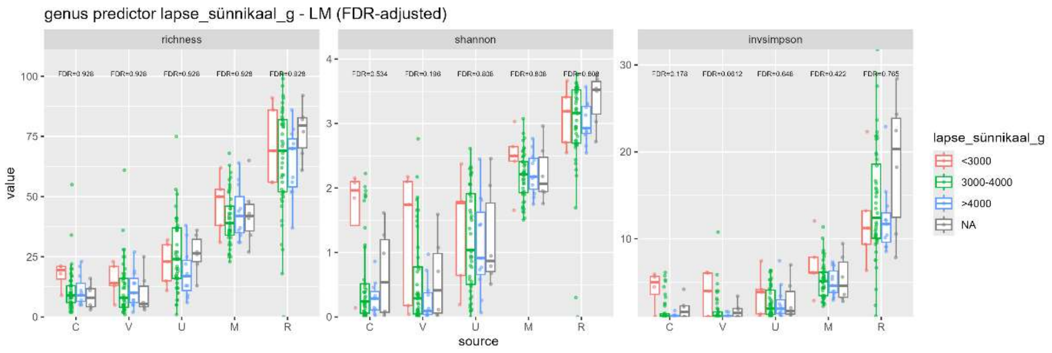

Species

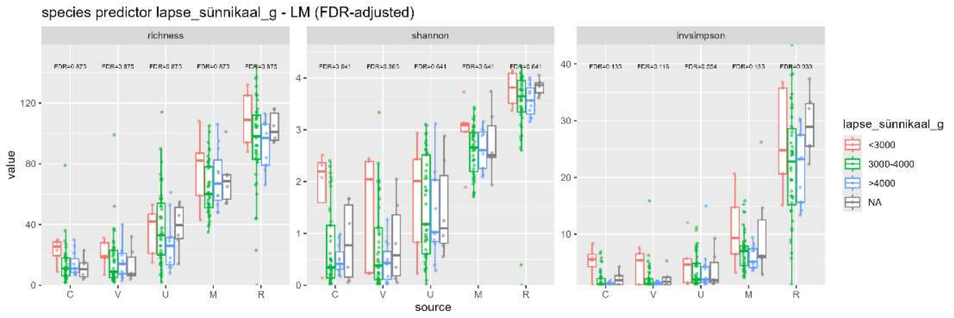

Duration of pregnancy at delivery (w)

Genus

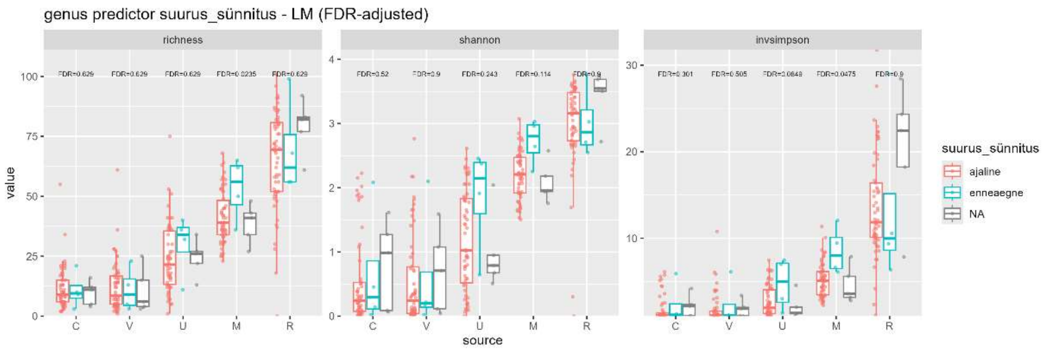

Species

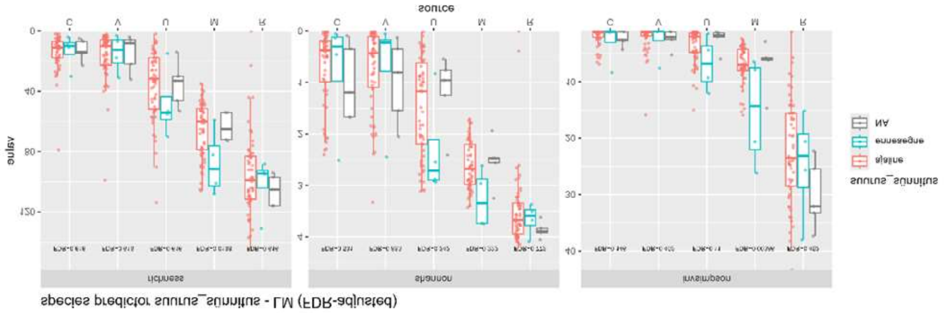

Delivery mode

Genus

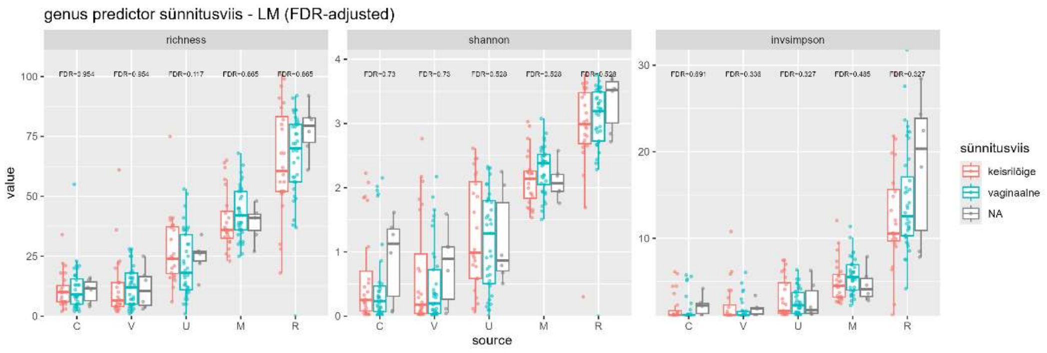

Species

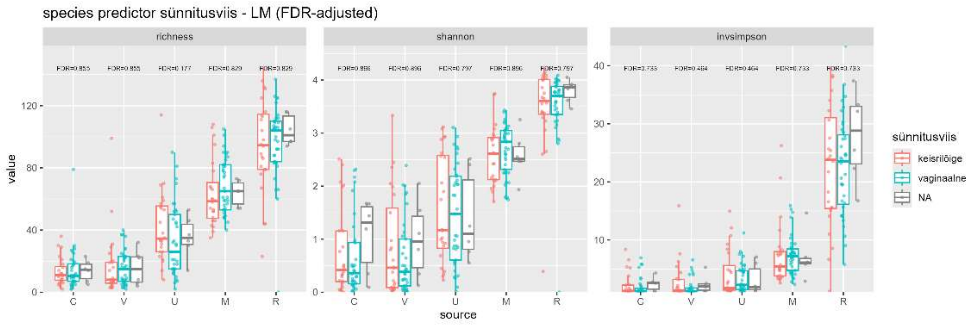

Supplement: Supplementary file 1 [file DataSheet1.pdf]
